# Fragments of gD Protein as Inhibitors of BTLA/HVEM Complex Formation - Design, Synthesis, and Cellular Studies

**DOI:** 10.3390/ijms21228876

**Published:** 2020-11-23

**Authors:** Katarzyna Kuncewicz, Claire Battin, Adam Sieradzan, Agnieszka Karczyńska, Marta Orlikowska, Anna Wardowska, Michał Pikuła, Peter Steinberger, Sylwia Rodziewicz-Motowidło, Marta Spodzieja

**Affiliations:** 1 Faculty of Chemistry, University of Gdańsk, 80-308 Gdańsk, Poland; katarzyna.kuncewicz@ug.edu.pl (K.K.); adam.sieradzan.ug@gmail.com (A.S.); agnieszka_karczynska@wp.pl (A.K.); marta.orlikowska@ug.edu.pl (M.O.); s.rodziewicz-motowidlo@ug.edu.pl (S.R.-M.); 2 Division of Immune Receptors and T Cell Activation, Institute of Immunology, Medical University of Vienna, 1090 Vienna, Austria; claire.battin@meduniwien.ac.at (C.B.); peter.steinberger@meduniwien.ac.at (P.S.); 3 Department of Embryology, Laboratory of Tissue Engineering and Regenerative Medicine, Medical University of Gdańsk, 80-210 Gdańsk, Poland; anna.wardowska@gumed.edu.pl (A.W.); pikula@gumed.edu.pl (M.P.)

**Keywords:** B- and T-lymphocyte attenuator, herpes virus entry mediator, glycoprotein D, immune checkpoint inhibitors, peptides

## Abstract

One of the major current trends in cancer immunotherapy is the blockade of immune checkpoint proteins that negatively regulate the immune response. This has been achieved through antibodies blocking PD-1/PD-L1 and CTLA-4/CD80/CD86 interactions. Such antibodies have revolutionized oncological therapy and shown a new way to fight cancer. Additional (negative) immune checkpoints are also promising targets in cancer therapy and there is a demand for inhibitors for these molecules. Our studies are focused on BTLA/HVEM complex, which inhibits T-cell proliferation and cytokine production and therefore has great potential as a new target for cancer treatment. The goal of the presented studies was the design and synthesis of compounds able to block BTLA/HVEM interactions. For that purpose, the *N*-terminal fragment of glycoprotein D (gD), which interacts with HVEM, was used. Based on the crystal structure of the gD/HVEM complex and MM/GBSA analysis performed on it, several peptides were designed and synthesized as potential inhibitors of the BTLA/HVEM interaction. Affinity tests, ELISA tests, and cellular-based reporter assays were performed on these compounds to check their ability to bind to HVEM and to inhibit BTLA/HVEM complex formation. For leading peptides candidates, all-atom and subsequent docking simulations with a coarse-grained force field were performed to determine their binding modes. To further evaluate their potential as drug candidates, their stability in plasma and their cytotoxicity effects on PBMCs were assessed. Our data indicate that the peptide gD(1-36)(K10C-T29C) is the best candidate as a future drug. It interacts with HVEM protein, blocks the BTLA/HVEM interaction, and is nontoxic to cells. The present study provides a new perspective on the development of BTLA/HVEM inhibitors that disrupt protein interactions.

## 1. Introduction

The B- and T-lymphocyte attenuator (BTLA) and herpesvirus entry mediator (HVEM) are immune checkpoint proteins (ICPs), which regulate the endogenous immune response against tumors [1,2]. The BTLA/HVEM complex, in a similar way to PD-1/PD-L1 [3] and CTLA-4/CD80/CD86 [4], provides inhibition of T-cells in the tumor microenvironment. Overexpression of BTLA/HVEM has been described in gastric cancer [5], bladder cancer [6], hepatocellular carcinoma [7], and melanoma [8]. Two monoclonal recombinant, humanized anti-BTLA antibodies were recently approved by the US Food and Drug Administration (FDA) for clinical trials [9,10], but a search for new molecules able to block BTLA/HVEM interactions is still necessary.

HVEM is a transmembrane glycoprotein [11], which can interact with several proteins [12]. HVEM acts as a receptor for lymphotoxin α (LTα) and LIGHT protein [13], and as a ligand for the BTLA [2] and cluster of differentiation 160 (CD160) [14]. LIGHT stands for “homologous to lymphotoxin, exhibits inducible expression, and competes with HSV glycoprotein D for herpes virus entry mediator, a receptor expressed on T lymphocytes”. The BTLA/HVEM or CD160/HVEM interaction delivers a co-inhibitory signal to T-cell activation, while the HVEM/LIGHT or HVEM/LTα binding leads to a co-stimulatory signal [1,2,15,16]. HVEM belongs to the tumor necrosis factor (TNF) receptor superfamily, and its extracellular fragment consists of four cysteine-rich domains (CRDs), each containing about 40 amino acid residues [17,18]. The CRD2 and CRD3 domains are involved in the binding of LTα and LIGHT [19], while CRD1 interacts with BTLA [12] and CD160 [14,20]. HVEM, using the CRD1 domain, also binds glycoprotein D (gD), which allows herpes simplex virus-1 and -2 (HSV-1 and HSV-2) to initiate infection via multiple entry routes [21]. The gD/HVEM interaction has been previously studied, particularly in the context of viral diseases. It was shown that soluble forms of gD effectively inhibit viral entry of HSV-1 and HSV-2 into cultured cells, and the whole gD protein and/or its N-terminal fragments could be used in herpesvirus vaccination [22,23]. Some reports also highlight the role of gD protein in modulating immune responses in cancer. Glycoprotein D disrupts BTLA/HVEM complex formation thereby presumably mediating immune activation by promoting interaction of HVEM with LIGHT and/or LTα [24,25].

BTLA and gD proteins belong to the immunoglobulin (Ig) superfamily, but glycoprotein D consists of a variable domain (IgV) [26] while BTLA possesses an intermediate domain (IgI) [12]. BTLA interacts with HVEM using the fragments of the Ig domain while gD binds to HVEM through an N-terminal loop that extends from the Ig core [12,26]. Comparison of the crystal structure of BTLA/HVEM complex (PDB: 2AW2) with gD/HVEM complex (PDB: 1JMA) shows that the BTLA and gD binding sites on HVEM largely overlap and involve similar structural motifs. In both complexes, the β-strands created by BTLA or gD form antiparallel β-sheets with β-structures present in HVEM. The fragments of BTLA or gD which interact with HVEM possess different amino acid sequences but for both complexes - BTLA/HVEM and gD/HVEM - the backbone hydrogen bonds in the main chains are crucial for forming the intermolecular β-sheet [12,26,27].

Formation of the gD/HVEM complex requires structural changes in the gD protein. In this process, the C-terminal part of gD protein detaches and the residues 1 to 22 of gD form the intramolecular β-hairpin structure with residues 23 to 36 [28,29,30]. The role of gD protein conformation during the interaction with HVEM was previously studied by Stump and Sticht. Using molecular dynamics simulations, they showed that the mutation Q27A changes the conformation of gD protein (particularly the orientation of M11 and R18) and the protein adopts a conformation with reduced HVEM-binding capacity [30]. It was also shown that a mutant of gD with a disulfide bond stabilizing the N-terminus of the protein has a higher affinity to HVEM than the wild type protein [28]. All this data indicates that the conformation of gD is crucial for binding of proteins and even small conformational changes in the N-terminus of gD could significantly change the affinity of the protein to HVEM.

The aim of the presented studies was to design BTLA/HVEM immune checkpoint inhibitors that could increase the immune response against tumors by blocking BTLA inhibition. Based on the amino acid sequence of the gD protein, we generated peptides able to mimic the most important binding patterns of BTLA and target the appropriate residues in HVEM. To design the inhibitors the crystal structure of the gD/HVEM complex and molecular mechanics generalized Born surface area (MM/GBSA) analysis were taken into consideration. The ability of the peptides to interact with HVEM was confirmed using affinity tests, and their inhibitory properties were studied using enzyme-linked immunosorbent assays (ELISA). In addition, the peptides were characterized in a functional in vitro assay based on a cellular reporter platform. For the most promising compounds, their stability in plasma and their effect on proliferation of human peripheral mononuclear blood cells (PBMCs) were studied to check their potential as drug candidates.

## 2. Results

**Figure 1:**
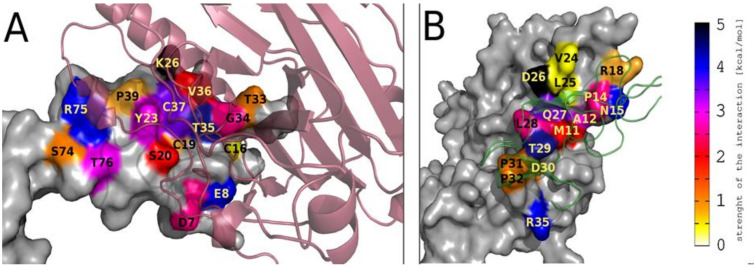
Representations of the most important HVEM (**A**) and gD (**B**) amino acid residues involved in gD/HVEM complex formation, based on the pairwise per-residue energy decomposition method. In Figure A, the HVEM structure is represented by surface area, and the gD structure is shown in cartoon representation (semi-transparent, dark red). In Figure B, the gD structure is represented by surface area, and the HVEM structure is shown in cartoon representation (semi-transparent, green). Surface areas of important residues are colored according to the energy scale (shown on the side).

For a better understanding of the interaction between gD and HVEM protein, all-atom molecular dynamics simulations (MD) of gD/HVEM complex (PDB code: 1JMA) were performed. Before running MD simulations, we modeled two amino acid loop regions in HVEM (D93 and G94) using Pymol building tools, as those residues are missing in the crystal structure. To estimate the contribution of individual amino acid residues from gD to the binding affinity with HVEM, MM/GBSA calculations were performed [31,32]. Thus, we analyzed the pairwise per-residue energy decomposition (Figure S1A) and the fraction of contacts formed (Figure S1B) between gD and HVEM in the complex. In addition, the per-residue energy decomposition was analyzed to investigate the total contribution of each amino acid of HVEM (Figure S2A) and gD (Figure S2B) molecules in the gD/HVEM binding. Finally, by comparing the pairwise per-residue and the per-residue energy decomposition methods, the key residues in gD and HVEM are highlighted in Table 1. On the basis of the obtained results, it can be seen that the M11-R35 fragment of the gD protein and the D7-E8, C16-P39, and S74-T76 fragments of HVEM protein are involved in the interaction of both proteins. Figure 1 shows the structural representation of the most important gD and HVEM residues involved in the gD/HVEM interaction, obtained from MM/GBSA energy decomposition analysis. It should be noted that strong interactions between gD and HVEM (blue and black areas) are well distributed on their contact surface (HVEM: E8, T35, R75; gD: N15, D26, T29, R35), which suggests very stable binding.

MM/GBSA analysis and the crystal structure of gD/HVEM enable us to determine the most important amino acid residues for protein interactions (Table 1). Then we compared our data with the experimental studies previously published by Connolly et al. All results are in agreement and highlight that the amino acids M11, N15, L25, Q27, L28, T29, and D30 in gD are crucial for binding to HVEM and that substitution of one of them causes a lack of interaction between gD and HVEM protein [27]. Moreover, all the aforementioned amino acids in gD protein (except N15) interact with the CRD1 domain of HVEM (Table S1). MM/GBSA analysis also indicated that A12, P14, R18, V24, D26, P31, P32, R35 in gD are conducive to gD/HVEM interaction. For instance, D26 and R35 in gD and K26 and E8 in HVEM form one of the strongest interactions in the gD/HVEM complex (Table S1). Some residues were expected to have a strong or moderate destabilizing effect on the formation of the gD/HVEM complex, however, the MM/GBSA analysis revealed that all amino acid residues are conducive or neutral to the proteins’ interactions. A very slight destabilizing effect was observed only for D6, K10, and D13 (Figure S2).

### 2.1. Peptide Design

**Figure 2:**
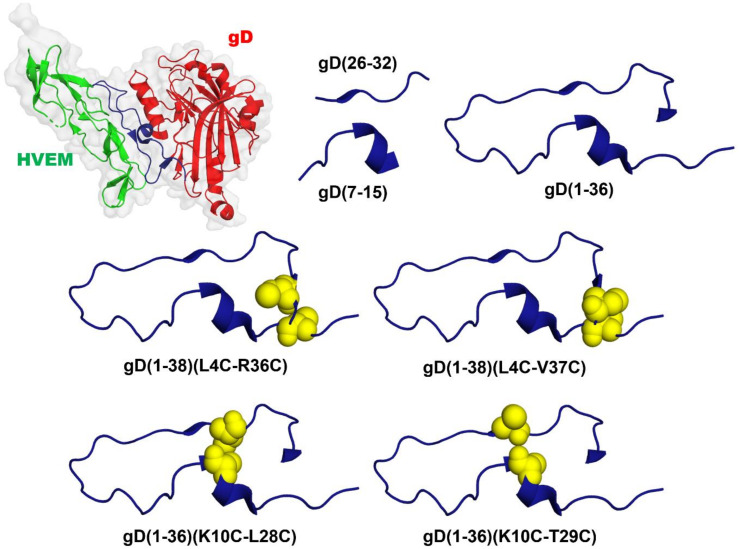
The crystal structure of gD/HVEM complex (PDB: 1JMA) with marked N-terminal fragment of the gD protein based on which potential inhibitors were designed (HVEM, green; gD, red; N, terminal fragment of the gD protein based on which peptides were designed, dark blue; cysteine residues forming additional disulfide bonds in designed peptides are marked as yellow spheres).

Based on the MM/GBSA analysis results (Table 1 and Figure 1), and the interaction between proteins in the crystal structure of the gD/HVEM complex (interactions between β-structures of both proteins), we designed and synthesized several peptides, which were fragments of gD protein, as potential inhibitors of BTLA/HVEM complex formation. The amino acid sequences of the peptides are presented in Table 2. The first two peptides, namely gD(7-15) and gD(26-32), contain the amino acids defined as “hot spots” in gD protein. The third peptide, gD(1-36), joins the two fragments (7-15 and 26-32) crucial for the gD/HVEM interaction. The other four peptides contain the amino acids, which are most essential in gD for interaction with HVEM and additionally have cysteine residues that form disulfide bridges and ensure the formation of the β-hairpin structure in the designed peptides, similar to the N-terminus of gD protein (Figure 2).

### 2.2. Affinity Studies

Affinity tests were performed to confirm the interaction of the designed peptides with HVEM protein. The peptide was added to a microcolumn with immobilized HVEM protein, and binding was checked using mass spectrometry, according to a procedure described earlier [33]. The results showed that the short and linear peptides: gD(26-32) and gD(7-15) do not interact with HVEM protein (not present in the elution fraction) while the longer or cyclic peptides bind to HVEM (signals m/z present in the elution fraction). The results are presented in Table 3.

### 2.3. ELISA Tests for BTLA/HVEM and HVEM/LIGHT Complexes

**Figure 3:**
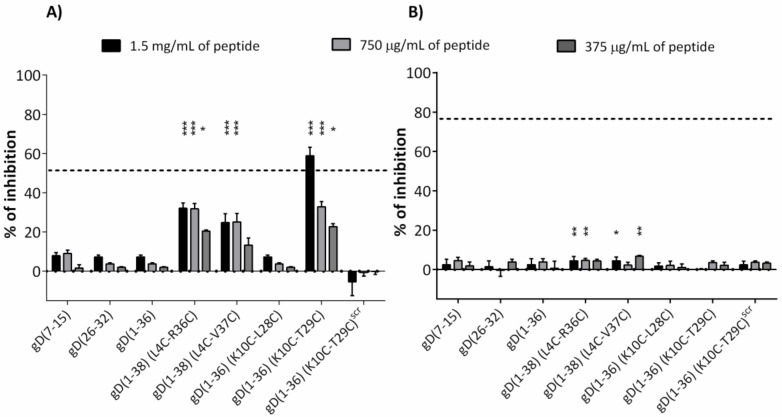
The inhibitory properties of the peptides for the formation of (**A**) BTLA/HVEM (**B**) HVEM/LIGHT complexes, determined by ELISA. The grey and dotted back lines correspond to the percentages of inhibition observed with an anti-HVEM blocking antibody (Mean +/-SD). Statistical analysis was performed using one-way analysis of variance (ANOVA) followed by Dunnet’s post-hoc test. ***: *p* < 0.001, **: *p* < 0.01, *: *p* < 0.05.

ELISA tests were performed to check the inhibitory properties of the peptides. HVEM protein was first immobilized in microplates and then the peptides were added in three different concentrations and incubated with HVEM. Unbound peptides were then removed and BTLA-Fc protein was added. For the detection of BTLA-Fc protein, a goat anti-human IgG (H+L)-HRP conjugate and TMB (3,3’,5,5’-tetramethylbenzidine) were applied. As a positive control, anti-HVEM antibodies, which inhibit the BTLA/HVEM complex formation by about 50%, were used (Figure 3A and Figure S3). A titration assay showed that the best inhibitory properties were observed for gD(1-36)(K10C-T29C). This peptide significantly blocks the binding of HVEM to BTLA, whereas the scrambled peptide gD(1-36)(K10C-T29C)^SCR^ had no effect (the amino acid sequence of the peptide is given in Table S2). The other peptides showed less or no ability to inhibit BTLA/HVEM complex formation (Figure 3A). The inhibitory properties of the peptides were also studied for the complex HVEM/LIGHT, using the same procedure and conditions as described above, but with the application of LIGHT protein instead of BTLA protein. A significant increase in inhibitory properties was observed only for the anti-HVEM antibodies (positive control), which inhibited the HVEM/LIGHT binding by about 75% (Figure 3B and Figure S3). None of the tested peptides inhibited the formation of the LIGHT/HVEM complex (Figure 3B).

### 2.4. Evaluation of the Inhibitory Properties of gD Peptides in a Cellular Reporter System

**Figure 4:**
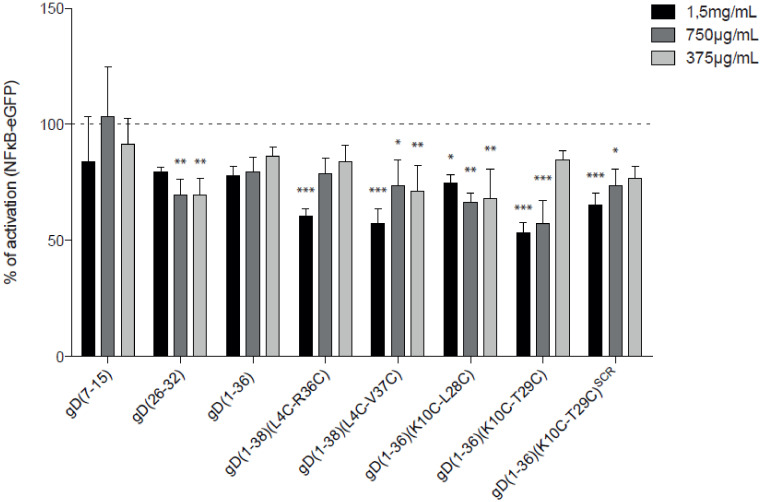
The inhibitory function of the peptides in a reporter cell-based assay. HVEM reporter cells were stimulated with TCS expressing BTLA in the absence or presence of the indicated gD peptides at concentrations of 1.5 mg/mL, 750 μg/mL, and 375 μg/mL. Reporter gene expression (NFκB-eGFP), upon stimulation with TCS-BTLA, was normalized to reporter activation after stimulation with TCS control in the presence of the respective peptides. BTLA/HVEM stimulation in the absence of peptides was set to 100% activation. Results are shown for three experiments performed independently in duplicate. Data are depicted as mean with SEM. * indicates statistically significant differences compared to full activation (100%), two-way ANOVA followed by Bonferroni’s post hoc test; *p* < 0.0001.

To assess the capacities of the gD peptides to interfere with the BTLA/HVEM complex in a functional in vitro setting, reporter cells (NFκB-eGFP), which are based on the human Jurkat T cell line JE6.1 were transduced to express HVEM [34]. These reporter cells can be stimulated by T cell stimulator cells (TCS) expressing a membrane-bound anti-CD3 antibody fragment, which is able to engage the TCR-CD3 complex and thereby activate the NFκB pathway [35]. In addition, to control TCS, TCS expressing the corresponding co-stimulatory ligand BTLA were generated in order to trigger HVEM in trans on the reporter cells (Figure S4). To validate the HVEM reporter system, control, and HVEM - expressing reporter cells were stimulated with control TCS and TCS expressing BTLA (Figure S4). Engagement of BTLA with HVEM induced high expression of NFκB-eGFP. To determine the inhibitory properties of the gD peptides, HVEM reporter cells were pre-incubated with the indicated peptides at different concentrations, followed by stimulation with control TCS or TCS-BTLA (Figure 4). The peptides gD(1-38)(L4C-R36C), gD(1-38)(L4C-V37C), and gD(1-36)(K10C-T29C) had the strongest capacity to interfere with the BTLA/HVEM complex at a concentration of 1.5 mg/mL, as shown by a reduction of NFκB-eGFP activation (Figure 4). These peptides also had a dose-dependent effect on blocking HVEM. A weak blocking effect was observed for gD(26-32), gD(1-36), gD(1-36)(K10C-L28C), and gD(1-36)(K10C-T29C)^SCR^, while no effect was seen for gD(7-15) (Figure 4).

### 2.5. Stability of the Peptides in PBS, Cell Culture Medium, and Plasma

A low stability in solution, which could be connected with many different processes such as aggregation, conformation changes, and chemical degradation, including deamidation and isomerization, oxidation, hydrolysis, and racemization, is a major concern for the therapeutic application of peptides [36]. For the presented study, the stability of the peptides in PBS buffer and medium (solutions used in tests described above) was studied and determined using RP-HPLC. The analysis was done by comparing the area under the peaks in a control sample (peptide dissolved in water, time = 0) and a sample after incubation in PBS or medium. All peptides were stable in PBS, and only small degradation over time was observed (Figure S5A). In the medium, gD(7-15) peptide was almost completely degraded after 24 h, while gD(1-36) was about 50% degraded (Figure S5B). The peptides which showed the best inhibitory properties in ELISA and the reporter assays, namely gD(1-38)(L4C-R36C), gD(1-38)(L4C-V37C), and gD(1-36)(K10C-T29C), were stable under all tested conditions and only a slight reduction in their amount was observed over time.

Peptides with the potential to be drugs could also be susceptible to enzymatic degradation by endogenous proteases present in human blood. Stability studies in plasma, obtained as supernatants after centrifugation of blood supplemented with anticoagulants [37], were performed only for the three peptides, namely gD(1-38)(L4C-R36C), gD(1-38)(L4C-V37C) and gD(1-36)(K10C-T29C), which showed the best effect in ELISA assays and the cellular reporter system as inhibitors of BTLA/HVEM complex formation. The procedure was the same as described previously. A significant decrease in the peptide concentration in plasma was observed at time = 0, but additional signals providing evidence of peptide degradation were not detected for gD(1-38)(L4C-R36C) and gD(1-36)(K10C-T29C) (Figures S6 and S7A,C). For the gD(1-38)(L4C-V37C) peptide, a small degradation over time was noticed (Figures S6 and S7B). Those results indicate that the peptides are bound by some plasma components. 45%, 13%, and 38% of the initial concentration of the peptide in plasma was observed at time = 0 for gD(1-38)(L4C-R36C), gD(1-38)(L4C-V37C), and gD(1-36)(K10C-T29C), respectively. After 24 h an additional reduction in the amount of peptide to a level of 21%, 11%, and 20% for gD(1-38)(L4C-R36C), gD(1-38)(L4C-V37C), and gD(1-36)(K10C-T29C), respectively, was noticed (Figure S6).

### 2.6. XTT Cell Proliferation Assay

**Figure 5:**
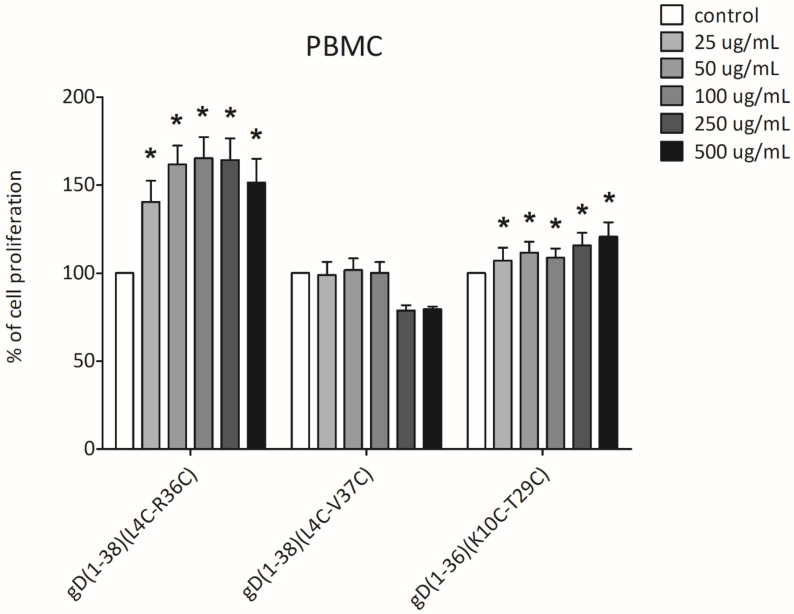
The effect of selected gD peptides on the proliferation of PBMCs. The graph shows results from 3 independent experiments (4 replicates in each, *n* = 12). Results are presented as mean with SD. * indicates statistically significant differences compared to control (unstimulated PBMCs), Mann–Whitney U test, *p* < 0.005.

An XTT proliferation assay was used to evaluate the impact of the best peptide inhibitors of BTLA/HVEM complex formation on the proliferation of peripheral blood mononuclear cells. This assay is widely utilized in the measurement of a broad spectrum of biologically active compounds, in order to assess their impact on cell proliferation and, thus, indirectly, cell cytotoxicity [38,39]. The effect of gD(1-38)(L4C-R36C), gD(1-38)(L4C-V37C), and gD(1-36)(K10C-T29C) on cell proliferation, and indirectly cell cytotoxicity, was examined in PBMCs from healthy volunteers. 24 h stimulation with gD(1-38)(L4C-R36C) and gD(1-36)(K10C-T29C) revealed no negative effects in terms of cell cytotoxicity. The only exception was gD(1-38)(L4C-V37C), which caused a small decrease in PBMC proliferation at the highest concentrations used (500 and 250 µg/mL). The other concentrations of gD(1-38)(L4C-V37C) had no impact on PBMC proliferation, as the observed absorbance did not exceed the values obtained in control samples (unstimulated PBMCs). The other two compounds, gD(1-38)(L4C-R36C) and gD(1-36)(K10C-T29C), revealed a rather opposite effect, as the proliferation was significantly increased. The proliferation rate of PBMCs stimulated with gD(1-38)(L4C-R36C) even exceeded 150% over a concentration range of 50-500 µg/mL. In the case of gD(1-36)(K10C-T29C) this effect was less spectacular, as the proliferation of PBMC was enhanced by about 25% (at 250 and 500 µg/mL) (Figure 5).

### 2.7. Flexibility Studies and Docking Simulations of Leading Peptides

**Figure 6:**
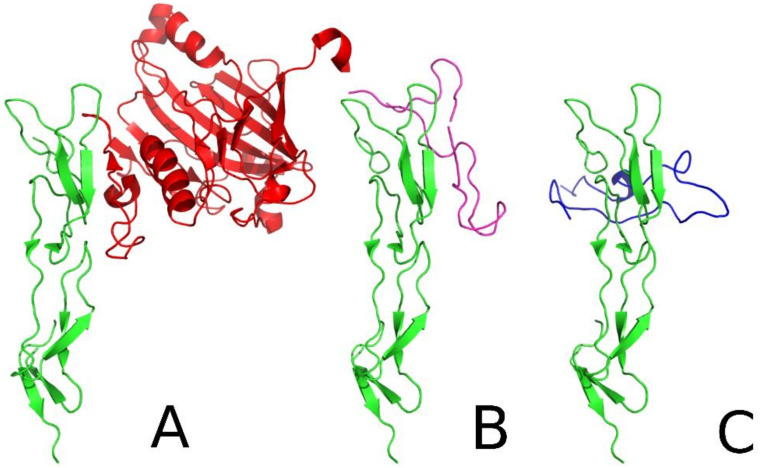
Comparison of the crystal structure of the gD (red)/HVEM (green) complex (**A**) with the structures of the fourth cluster centroid for gD(1-38)(L4C-V37C) (purple)/HVEM (green) complex obtained after docking simulation (**B**) and the first cluster centroid for the gD(1-36)(K10C-T29C) (dark blue)/HVEM(green) complex obtained after docking simulation (**C**).

The peptides that showed the best inhibitory properties were also studied using molecular modeling methods. The all-atom simulations of the three leading peptides, gD(1-38)(L4C-R36C), gD(1-38)(L4C-V37C), and gD(1-36)(K10C-T29C), revealed large flexibility in these compounds (Figure S8). The comparison between the peptide structure after all-atom simulation and the crystal structure of gD corresponding to the peptide fragment revealed RMSD values of 8.26 Å, 6.00 Å, and 7.49 Å for peptides gD(1-38)(L4C-R36C), gD(1-38)(L4C-V37C), and gD(1-36)(K10C-T29C), respectively. These peptide structures were docked to HVEM protein using an UNRES force field. As a result, the gD(1-38)(L4C-V37C) and gD(1-36)(K10C-T29C) peptides revealed the same binding site to HVEM as the crystal structure of gD (Figure 6A). For the gD(1-36)(K10C-T29C) peptide (Figure 6C), the same binding site as in gD was observed for the dominant cluster. The dominant cluster is the one with the highest probability, and the higher the probability of the cluster sharing a similar binding site, the stronger the peptide binds. This indicated that gD(1-36)(K10C-T29C) peptide strongly interacts with HVEM protein. The RMSD of the simulated complex to the crystal-like structure was 7.74 Å, which is similar to the RMSD of the non-bound peptide, indicating some conformational adaptation of the peptide to the HVEM binding site. The probability of this cluster was 14.4%. In the case of gD(1-38)(L4C-V37C), the fourth most probable cluster revealed the same binding site as in gD. The RMSD of the complex was 6.98 Å, which is similar to the RMSD of the unbound peptide. The probability of this cluster was 9.5%, which indicates significantly weaker binding to HVEM (Figure 6B). However, for gD(1-38)(L4C-R36C), no binding site similar to that in the gD/HVEM crystal structure was observed (the binding of the peptide to HVEM was random). This might indicate that gD(1-38)(L4C-R36C) peptide is not a competitive inhibitor of gD/HVEM complex formation.

In the crystal structure, gD binding and the HVEM binding β-sheets form a small angle. The conformational analysis revealed that the angle between the β-sheets of gD(1-38)(L4C-V37C) was more parallel to the HVEM chain than found in the crystal structure, while the β-sheets in the gD(1-36)(K10C-T29C)/HVEM complex are perpendicular. In all the presented complexes (Figure 6), similar residues from HVEM protein are involved. 5K, 7D-9Y, 16P-23Y, 30G-35T, 37C, 39P-40C, 75R-76T are common fragments from HVEM protein within 8Å from the binding partner.

## 3. Discussion

The rational design of the peptides or peptidomimetics, which are potential inhibitors of protein binding, based on their ligand structure, has been successfully applied in many studies. Such compounds interact with receptor/ligand interactions and disrupt protein complex formation. In the area of immune checkpoints, this approach has been used to design inhibitors of PD1/PD-L1 [40,41,42], CTLA-4/CD80/CD86 [43], and BTLA/HVEM [33,44] complex formation.

The rational design of the peptides or peptidomimetics, which are potential inhibitors of protein binding, based on their ligand structure, has been successfully applied in many studies. Such compounds interact with receptor/ligand interactions and disrupt protein complex formation. In the area of immune checkpoints, this approach has been used to design inhibitors of PD1/PD-L1 [40,41,42], CTLA-4/CD80/CD86 [43], and BTLA/HVEM [33,44] complex formation.

HVEM is a very challenging target since it is part of a complex network of receptors and ligands. Some of these interaction function to stimulate immune responses (LIGHT, LTα) [15,45], whereas the interaction with BTLA and CD160 mainly act inhibitory [1,14]. HVEM also has two spatially distinct ligand-binding regions, one (CRD2 and CRD3) for LIGHT and LTα [19] and a second (CRD1) for BTLA [12], CD160 [20], and gD [27]. That feature of the protein is very useful in the design of compounds able to block the negative function of HVEM while maintaining its stimulating properties. In the presented study, we used the amino acid sequence of gD protein, which interacts mainly with the CRD1 domain of HVEM, to design inhibitors of BTLA and HVEM interactions. We focused on gD protein, since it has been previously demonstrated that it could disturb BTLA/HVEM binding [25].

In the presented study, we used the N-terminal fragment of the gD protein to design inhibitors of BTLA/HVEM interactions. We first studied three linear peptides gD(7-15), gD(26-32), and gD(1-36). The first two, according to the crystal structure of gD/HVEM, are the binding sites of gD protein and are directly involved in the interaction with HVEM [27]. The third peptide consists of all amino acids present in the β-hairpin created by the N-terminus of gD protein. Peptides gD(7-15) and gD(26-32) do not interact with HVEM protein and do not inhibit BTLA/HVEM complex formation. The peptide gD(1-36) binds to HVEM, but did not prevent protein interaction in ELISA. These data are in agreement with our predictions because those peptides are too short and/or flexible to adopt a similar conformation as a gD-binding fragment, to mimic the most important binding patterns of BTLA and to target the appropriate residues in HVEM. However, gD(26-32) and gD(1-36) showed weak blocking effects in the cellular reporter assay, as evidenced by a reduction in HVEM activation.

Taking into consideration the conformation of gD protein in a complex with HVEM protein, we designed two other peptides gD(1-38)(L4C-R36C) and gD(1-38)(L4C-V37C), which are stabilized by disulfide bridges. The amino acids L4 and R36/V37 substituted by cysteines are situated directly opposite each other in the gD/HVEM crystal structure, and their side chains are oriented in the same direction (Figure 2). The disulfide bonds should stabilize the structure of these peptides and impose on them an intramolecular β-hairpin structure. In addition, MM/GBSA analysis indicated that the substituted amino acids are not crucial for the gD/HVEM interactions. The peptides bind to HVEM in affinity tests and possess some inhibitory properties in ELISA tests (about 30–35% at the highest peptide concentration; Figure 3A). For the peptides gD(1-38)(L4C-V37C) and gD(1-38)(L4C-R36C), inhibitory effects were also observed in the cellular reporter assays and they were able to block BTLA/HVEM interactions.

We also studied two peptides, gD(1-36)(K10C-L28C) and gD(1-36)(K10C-T29C), with substituted the amino acids crucial for the protein binding, L28 and T29, respectively. MM/GBSA indicated that L28 in gD interacts with V36 and T35 in HVEM, and for each pair, the interaction energy is about –2 kcal/mol (Table S1). On the other hand, T29 binds to T35 and P17 in HVEM, however, the first interaction is more significant with an energy of about –4 kcal/mol (Table S1). The pairs of amino acids L10-L28 and L10-T29 are situated directly opposite each other and should adopt a structure similar to the N-terminus of gD (Figure 2). The affinity studies show that both peptides interact with HVEM, but only one of them, gD(1-36)(K10C-T29C), significantly inhibits BTLA/HVEM binding in ELISA tests and reveals strong binding to HVEM in theoretical studies. In the cellular assays, gD(1-36)(K10C-T29C) greatly inhibited HVEM activation in a dose-dependent manner while the peptide gD(1-36)(K10C-L28C) only displayed small inhibitory properties. This suggests that the T29 residue is not crucial for binding of this peptide to HVEM, in contrast to the binding of gD protein to HVEM. The literature data show that the mutants T29P, L28P, L28G, and L28A completely abolish gD/HVEM interactions [27,46].

The blocking capacity of all peptides for the HVEM/LIGHT complex was also studied using ELISA. The peptides did not block the HVEM/LIGHT interaction, which shows that only the negative pathway of HVEM is inhibited, and the stimulating effect of the protein is preserved. That could be one of the most important advantages of peptides compared to antibodies, as HVEM antibodies block both functions of HVEM protein, which was confirmed by ELISA tests (Figure S3).

For the most promising peptides, additional studies were performed and their stability in human plasma and effect on PBMC proliferation were evaluated. The results confirmed that the peptides are stable in plasma, but their concentration rapidly decreases. It is probable that they interact with human serum albumin (HSA), which is a natural transporter of many compounds in the blood and possesses extraordinary ligand binding properties [47]. This fact is very important in terms of peptide distribution in blood and could be used to improve their delivery to cancer cells, but this issue requires more detailed studies. PBMC proliferation tests showed that two peptides, namely gD(1-38)(L4C-R36C) and gD(1-36)(K10C-T29C), stimulate cell proliferation and are not cytotoxic at any concentrations tested. The third peptide, gD(1-38)(L4C-V37C), had a small/minor negative effect on PBMCs proliferation at the highest concentration used.

In summary, all data indicate that the peptide gD(1-36)(K10C-T29C) is the best potential candidate as a future drug to block the BTLA/HVEM interaction. Molecular dynamics studies show that it interacts with HVEM more strongly than the other peptides and binds to HVEM in the same place as gD and BTLA protein. In addition, it is stable in human plasma and increases PBMC proliferation. This peptide could be the lead structure in the design of new compounds, which are inhibitors of BTLA/HVEM interactions. However, further studies are needed to determine its immunological potential, especially in the context of cancer treatment.

## 4. Materials and Methods

### 4.1. Sources of Proteins

The recombinant human HVEM with His tag protein was purchased from ACROBiosystems, Newark, DE, USA (#HVM-H52E9). Recombinant human protein BTLA-Fc was purchased from Novoprotein, Fremont, CA, USA (#CD06), and recombinant human LIGHT-Fc was purchased from ACROBiosystems, Newark, DE, USA (#LIT-H5265). Mouse anti-human HVEM antibodies were purchased from Abnova, Taipei City, Taiwan (#H00008764-M01) and Bio-Rad, Hercules, CA, USA (#MCA6072GA).

### 4.2. MD Simulation Protocol

A complex structure (PDB: 1JMA), with a missing loop from the HVEM molecule (Asp93 and Gly94), rebuilt using Pymol building tools, was placed in an explicit solvent octahedral water box (TIP3PBOX) with a minimal distance of 10 Å between the complex and the box sides, and counter ions were then added to the system. The following disulfide bridges between cysteine residues in HVEM were declared: 4-15, 16-29, 19-37, 40-55, 58-73, 61-81, 83-100, and 89-97; and in gD the following disulfide bridges were declared: 66-189, 106-202, 118-127. The complex structure was minimized in the AMBER ff14SB force field [31] initially with positional Cα constraints of 10 kcal/(mol·Å^2^) (steepest descent for 1500 steps and conjugate gradient for 1000 steps), and then without restraints (steepest descent for 6000 steps and conjugate gradient for 3000 steps). After minimizations, the system was heated up from 0 to 300 K for 10 ps with positional restraints and then equilibrated with NPT MD simulations (constant number of particles, under constant pressure of 105 Pa and with a constant temperature of 300 K) without constraints for 0.5 ns. An 8 Å cutoff for nonbonded interactions and the particle mesh Ewald method for long-range electrostatic interactions were applied. MD simulations were performed using an ff14SB force field [48] for 200 ns under NPT conditions in explicit water TIP3P (with a SHAKE algorithm to perform bond length constraints where covalent bonds involving hydrogen are constrained), using Langevin dynamics (with the collision frequency equal to 1 ps^−1^) and Berendsen barostat for both systems.

### 4.3. Free Energy Calculations

The free energy calculations (binding free energy, ΔG_bind_) were performed with molecular mechanics generalized Born surface area (MM/GBSA) analysis, using the modified GB model with energy decomposition [32,49]. The energy was decomposed according to per-residue and pairwise per-residue schemes with local (between 1-4 atoms) electrostatic and local Van der Waals energies being taken into account.

### 4.4. Post-Processing of the MD Trajectory

Contact analysis of the MD trajectory was performed using the CPPTRAJ program [50], which was designed for processing coordinate trajectories and data files generated using the AMBER package. Contacts between HVEM and gD amino acid residues were traced as any heavy atom pair (i.e., any atoms except hydrogen atoms) closer than 4Å from each other.

### 4.5. Peptides Synthesis, Purification and Disulfide Bond Formation

Peptide synthesis was performed according to the standard protocol of solid-phase peptide synthesis using Fmoc/tBu chemistry. The synthesis was performed on a semiautomated peptide synthesizer (Millipore 9050 Plus PepSynthesizer, Millipore Corporation, Burlington, VT, USA) using TentaGel R RAM resin (0.19 mmol/g) [51]. Acetylation of the N-terminal amino group of the peptides was performed using 1-acetylimidazole (1.10 g/1 g of resin at room temperature for 24 h). During the synthesis, cysteine side chains were protected with triphenylmethyl (Trt) and then removed during cleavage of the peptides from the resin. The crude peptides were purified by RP-HPLC on a semipreparative Luna C8(2) column (20 × 250 mm, 5 µm, Phenomenex, Torrance, CA, USA). Before purification, the peptides were dissolved in H_2_O and a 10-fold excess of dithiothreitol (DTT) was added. The mixture was kept in an ultrasonic bath at 60 °C for 30 min. A linear gradient from 20% B in A to 60% B in A over 180 min was used (where A - H_2_O containing 0.1% TFA (v:v) and B - 80% acetonitrile in H_2_O containing 0.08% (v:v) TFA). The flow rate was adjusted to 15 mL/min and the separation process was monitored by UV absorbance at 222 and 254 nm. The purity of peptides was analyzed in a linear gradient from 5 to 100% B over 15 min by using RP-UHPLC and a Kinetex C8 column (2.1 × 100 mm, 2.6 μm, Phenomenex, Torrance, CA, USA). After purification, the peptide was dissolved in H_2_O and methanol (1:9, v:v), at a concentration of 40 mg/l, and the pH was adjusted and kept between 8 and 9 using ammonia. The solution was stirred at room temperature for 7 days, and compressed air was running through the solution. After this time, the solvents were evaporated and the peptides were lyophilized. Reaction progress was checked using analytical RP-UHPLC and LC-ESI-IT TOF MS (Shimadzu, Shimpol, Warsaw, Poland). After this process, the peptides were purified again using RP-HPLC and a Luna C18(2) column (250 × 4.6 mm, 5 μm, 100Å, Phenomenex, Torranca, CA, USA). A linear gradient from 30% B to 60% B over 100 min was used.

### 4.6. Affinity Tests

The affinity tests were performed according to the procedure described in our previous paper [33]. The MS spectrums were registered using a MALDI-TOF/TOF autoflex maX (Bruker, Billerica, MA, USA) mass spectrometer.

### 4.7. ELISA

A 96-well Immunograde plate (Brand, Wertheim, Germany) was coated with 5 μg/mL of recombinant HVEM-His protein in PBS (100 μL/well) and incubated overnight at 4 °C. The plate was washed 5 times (200 μL/well) with PBS-T (5 mM Na_2_HPO_4_, 150 mM NaCl with the addition of 0.3 M NaCl and 0.05% Tween-20, pH 7.4) and blocked with 5% BSA in PBS-T for 2 h at 37 °C. The peptides were then titrated in triplicate from 1.5 to 0.375 mg/mL (100 µL/well) in PBS-T and incubated for 24 h at 37 °C. Maximum binding of HVEM to BTLA/LIGHT was determined by adding PBS-T to negative control wells, whereas maximum inhibition was measured by adding anti-HVEM (1 µg/well). The plate was then washed and incubated with recombinant BTLA-Fc/LIGHT-Fc protein at 5 μg/mL in PBS-T (100 μL/well) for 2 h at 37 °C. Goat anti-human IgG (H+L)-HRP conjugate (Bio-Rad, Hercules, CA, USA) antibody was then added (1:3000; 100 μL/well) and incubated for 1 h at 37 °C. Finally, BTLA-Fc/LIGHT-Fc was detected by the addition of 100 μL/well of TMB (Thermo Scientific, Waltham, MA, USA). Absorbance was measured using Infinite M200 Pro (Tecan Life Sciences, Männedorf, Switzerland) at 650 nm and 492 nm. The percentage of inhibition was calculated with the assumption that PBS-T does not inhibit BTLA/HVEM or HVEM/LIGHT complex formation. ELISA tests were performed at least in triplicate. The data are presented as mean ± standard deviation (SD). The results were analyzed by one-way analysis of variance (ANOVA) followed by Dunnett’s post-hoc test using GraphPad Prism (San Diego, CA, USA).

### 4.8. Stability of Peptides in PBS

Each peptide was incubated in PBS at a concentration of 1 mg/mL. The incubation time points were 0, 2, 3, 6, and 24 h at 37 °C. Samples were then analyzed by RP-HPLC on a Kromasil C8 analytical column (250 mm, 4.6 mm, 5 μL) using a linear gradient from 5% B to 100% B in A in 60 min (for A and B: see purification of peptides). All stability tests were performed at least in triplicate.

### 4.9. Stability of Peptides in the Medium and Human Plasma

200 µL of medium (RPMI1640 with 10% FCS)/human plasma was added to 50 µL of each peptide (1 mg/mL). The samples were incubated for different times (0, 2, 3, 6, and 24 h) at 37 °C. They were then precipitated by the addition 1 mL of absolute ethanol, incubated on ice for 15 min and centrifuged at 18,000 rpm for 20 min at 4 °C. The supernatants were collected and dried under vacuum. The samples were then resuspended in 120 µL of 0.1% TFA and analyzed by RP-HPLC under the same conditions as described for the determination of the stability of the peptides in PBS. All stability tests were performed at least in triplicate. Blood was obtained from donor volunteers and centrifuged in lithium/heparin tubes. EDTA was used as an anticoagulant.

### 4.10. Cell Culture, Antibodies and Flow Cytometry

The Jurkat E6.1 cells and the mouse thymoma cell line BW5147 were derived from in-house stocks and cultured as previously described [52]. All cell lines were cultured in RPMI 1640 supplemented with 10% FBS, penicillin (100 U/mL), and streptomycin (100 μg/mL) (all from Sigma Aldrich, St. Louis, MO, USA). Cell lines were tested for mycoplasma contamination using a previously described reporter system [53]. The generation of the Jurkat NFκB-eGFP cell line and the T cell stimulator cell line (TCS) has been described previously [34,35]. The T cell stimulator cell line expresses a membrane-bound human CD3 antibody single-chain fragment, which can activate T cells by engaging their CD3/TCR complex [35]. To assess the surface expression of the receptors, the following antibodies were purchased from Biolegend (San Diego, CA, USA): HVEM-PE (122), BTLA-APC (MIH26), CD14-APC (63D3), and mCD45.2-APC (104). Flow cytometry analysis was performed on a FACSCalibur™ flow cytometer (BD Bioscience, Franklin Lakes, NJ, USA). FlowJo software (version 10.6.1, Tree Star, Ashland, OR, USA) was used for flow cytometry data analysis.

### 4.11. Cell-Based Reporter Assays

For functional assays, HVEM-expressing reporter cells (5 × 10^4^ cells/well) were pre-incubated with different concentrations of gD peptides for 30 min in a 96 flat bottom plate. Control TCS or TCS expressing BTLA (2 × 10^4^ cells/well) were subsequently added for 24 h at 37 °C with 5% CO_2_. After 24 h, the cells were harvested and stained with an mCD45.2 antibody to exclude TCS from the reporter cells. Expression of the reporter gene (eGFP) was then measured via flow cytometry. The mean and standard deviation of the gMFI of the viable population of reporter cells (mCD45.2+ stimulator cells were excluded) were determined. Each experiment was performed in duplicate.

### 4.12. UNRES Docking Procedure

To generate the starting structures, the in-house algorithm for semi-random placement of two molecules with respect to each other was used. Ligands were randomly rotated and placed in the proximity of receptors, as in our previous papers [44]. Multiplexed replica exchange molecular dynamics (MREMD) simulation was subsequently performed. Each MREMD docking simulation started from energy-minimization of generated semi-random orientations of molecules, which were subsequently subjected to ten million MD steps per replica, each of 4.89 fs of UNRES time. Twenty replicas were performed over a temperature range of 250 to 400 K, with two replicas at each temperature. The total time of the simulation reached 48.9 ns of UNRES time per replica, which equates to approximately 50 µs of real-time per replica due to smoothening of the energy landscape in the coarse-grained representation [54]. Replica exchanges were attempted every 10,000 steps, and snapshots were saved with the same frequency. The newest version of the scale-consistent UNRES force field [55] was used. After the simulation, the last 70% of the snapshots were analyzed by a weighted histogram analysis method (WHAM) [56] to generate the subset of most probable conformations at 300K. The clustering algorithm was then set to generate 10 groups of structures, and cluster centroids were produced.

### 4.13. XTT Cell Proliferation Assay

PBMCs were isolated from buffy coats (*n* = 3), obtained from blood donor volunteers, using histopaque density-gradient centrifugation (in accordance with the manufacturer’s instructions—Sigma Aldrich, St. Louis, MO, USA). After erythrocyte lysis, PBMCs were seeded on 96-well plates, at a density of 5 × 10^4^ cells/well, in RPMI 1640 medium supplemented with 10% of FBS and antibiotics. The compounds to be examined were then added at the following concentrations: 500, 250, 100, 50, and 25 µg/mL. After incubation for 24 h under appropriate conditions (37 °C for 4 h in the presence of 5% CO_2_), the reagent 2,3-Bis-(2-Methoxy-4-Nitro-5-Sulfophenyl)-2*H*-Tetrazolium-5-Carboxanilide (XTT) was added and the plates were incubated for a further 4 h (under the conditions described above), following the manufacturer’s instructions (Sigma Aldrich, St. Louis, MO, USA). The plates were then read using a standard plate reader at OD 490 nm. Cell proliferation was normalized with respect to an untreated control (100%) [57].

## Figures and Tables

**Table 1:** Key residues obtained from MM/GBSA energy decomposition analysis. Amino acid residues that reveal strong interaction in both methods of energy decomposition are highlighted.

Type of the Energy Decomposition	Amino Acid Residues of gD Involved in Important gD/HVEM Interactions	Amino Acid Residues of HVEM Involved in Important gD/HVEM Interactions
Per-residue	**M11, A12, P14, N15, V24, Q27, L28, T29, P31, P32, R35**	**P17, Y23, G34, T35, V36, C37, P39, R75, T76**
Pairwise per-residue	**M11, A12, P14, N15,** R18, **V24,** L25, D26, **Q27, L28, T29,** D30, **P31, P32, R35**	D7, E8, C16, **P17,** C19, S20, **Y23,** K26, T33, **G34, T35, V36, C37, P39,** S74, **R75, T76**

**Table 2:** The amino acid sequences of the designed peptides.

Peptide Name	Amino Acid Sequence
**gD(7-15)**	Ac-ASLKMADPN-NH_2_
**gD(26-32)**	Ac-DQLTDPP-NH_2_
**gD(1-36)**	Ac-KYALVDASLKMADPNRFRGKDLPVLDQLTDPPGVRR-NH_2_
**gD(1-38)(L4C-R36C)**	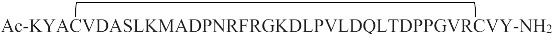
**gD(1-38)(L4C-V37C)**	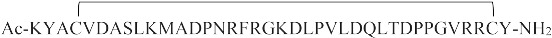
**gD(1-36)(K10C-L28C)**	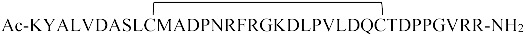
**gD(1-36)(K10C-T29C)**	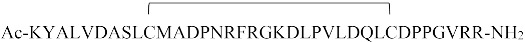

**Table 3:** Results of affinity tests. Three fractions were analyzed using mass spectrometry: supernatant, last wash, and elution. The binding of the peptide with the HVEM protein immobilized in the microcolumn was confirmed if the signals m/z (corresponding to the molecular weight of peptide) were present in the elution fraction and no signal was observed in the last wash fraction.

Peptide	[M+H]^+^_Calc._	Supernatant [M+H]^+^	Last Wash	Elution [M+H]^+^
gD(7-15)	987.12	987.48	-	-
gD(26-32)	825.86	848.32 [M+Na]^+^	-	-
gD(1-36)	4094.72	4094.88	-	4094.88
gD(1-38)(L4C-R36C)	4291.97	4291.37	-	4292.61
gD(1-38)(L4C-V37C)	4349.03	4349.20	-	4349.20
gD(1-36)(K10C-L28C)	4057.68	4057.11	-	4057.11
gD(1-36)(K10C-T29C)	4069.73	4070.37	-	4068.45

## Supplementary Materials

Supplementary materials can be found at https://www.mdpi.com/1422-0067/21/22/8876/s1. Figure S1. (A) interaction energies between gD and HVEM residues calculated by the energy decomposition on a pairwise per-residue level calculated using MM/GBSA analysis method. (B) the total fraction of contacts for the residue pair calculated using CPPTRAJ tool from AMBER package. Figure S2. The energy decomposition on per-residue basis for HVEM (A) and gD (B) amino acid residues. Figure S3. The inhibitory properties of the peptides were compared with two different commercially available anti-HVEM antibodies. Figure S4. Evaluation of HVEM in a reporter based system. Left panel: Cell surface expression of the indicated molecules on Jurkat NFκB-eGFP reporter cells and T cell stimulator cells (TCS) analysed via flow cytometry. Figure S5. The peptides stability (mean ± SEM) in A) PBS buffer, B) medium. Figure S6. The peptides stability (mean ± SEM) in plasma. Figure S7. Chromatogram comparison of A) gD(1-38)(L4C-R36C), B) gD(1-38)(L4C-V37C) and C) gD(1-36)(K10C-T29C) peptides before incubation (A), peptide and plasma t = 0 (B), plasma t = 0 (C), peptide and plasma t = 24 h (D), plasma t = 24 h (E). Figure S8. Structures of A) gD(1-38)(L4C-R36C), B) gD(1-38)(L4C-V37C) and C) gD(1-36)(K10C-T29C) obtained after all-atom simulations. Table S1. Description of the strongest interactions between gD and HVEM residues assessed based on the energy decomposition on a pairwise per-residue level calculated using MM/GBSA analysis method. Table S2. Amino acids sequences of gD(1-36)(L10C-T29C) and gD(1-36)(L10C-T29C)^SCR^ peptides.

## Abbreviations

BTLA	B- and T-lymphocyte attenuator
CD80/CD86	cluster of differentiation 80/86
CD160	cluster of differentiation 160
CRD	cysteine rich domain
CTLA-4	cytotoxic T lymphocyte antigen-4
DTT	dithiothreitol
ELISA	enzyme-linked immunosorbent assay
Fc	fragment crystallizable
FCS	fetal calf serum
FDA	U.S. Food and Drug Administration
gD	glycoprotein D
HVEM	herpes virus entry mediator
HSV	1 - herpes simplex virus type 1
HSV-2	herpes simplex virus type 2
ICP	immune checkpoint protein
Ig	Superfamily immunoglobulin-like superfamily
LIGHT	homologous to lymphotoxins
LTα	lymphotoxin α
MD	molecular dynamics
MM/GBSA	molecular mechanics Generalized Born surface area
MREMD	multiplexed replica exchange molecular dynamics
PBMC	peripheral blood mononuclear cell
PBS	phosphate-buffered saline
PD-1	programmed cell death 1
PDB	protein data bank
PD-L1	programmed cell death-ligand 1
RP-HPLC	reversed phase-high performance liquid chromatography
TCS	T cell stimulator cells
TMB	3,3’,5,5’-tetramethylbenzidine
TNF	tumor necrosis factor
UNRES	UNited RESidue
WHAM	weighted histogram analysis method
XTT	2,3-Bis-(2-Methoxy-4-Nitro-5-Sulfophenyl)-2H-Tetrazolium-5-Carboxanilide
