# Supplementary material for: Fragments of gD Protein as Inhibitors of BTLA/HVEM Complex Formation - Design, Synthesis, and Cellular Studies
Source: Int J Mol Sci. 2020 Nov 23;21(22):8876. doi: 10.3390/ijms21228876 (PMC7700651; doi:10.3390/ijms21228876)
Supplement: Supplementary file 1 [file ijms-21-08876-s001.pdf]

International Journal of Molecular Sciences

MDPI

Supplementary materials

Fragments of gD Protein as Inhibitors of BTLA/HVEM Complex Formation—Design, Synthesis, And Cellular Studies

Katarzyna Kuncewicz [1], Claire Battin [2], Adam Sieradzan [1], Agnieszka Karczyńska [1], Marta Orlikowska [1], Anna Wardowska [3], Michał Pikuła [3], Peter Steinberger [2], Sylwia Rodziewicz-Motowidło [1] and Marta Spodzieja[1,*]

[1] University of Gdańsk, Faculty of Chemistry, Wita Stwosza 63, 80-308 Gdańsk, Poland, katarzyna.kuncewicz@ug.edu.pl (K.K.); adam.sieradzan.ug@gmail.com (A.S.); agnieszka_karczynska@wp.pl (A.K.); marta.orlikowska@ug.edu.pl (M.O.); s.rodziewicz-motowidlo@ug.edu.pl (S.R.M.)

[2] Medical University of Vienna, Institute of Immunology, Division of Immune Receptors and T cell Activation, 1090 Vienna, Austria, claire.battin@meduniwien.ac.at (C.B.); peter.steinberger@meduniwien.ac.at (P.S.)

[3] Medical University of Gdańsk, Department of Embryology, Laboratory of Tissue Engineering and Regenerative Medicine, 80-210 Gdańsk, Poland, anna.wardowska@gumed.edu.pl (A.W.); pikula@gumed.edu.pl (M.P.)

* Correspondence: marta.spodzieja@ug.edu.pl

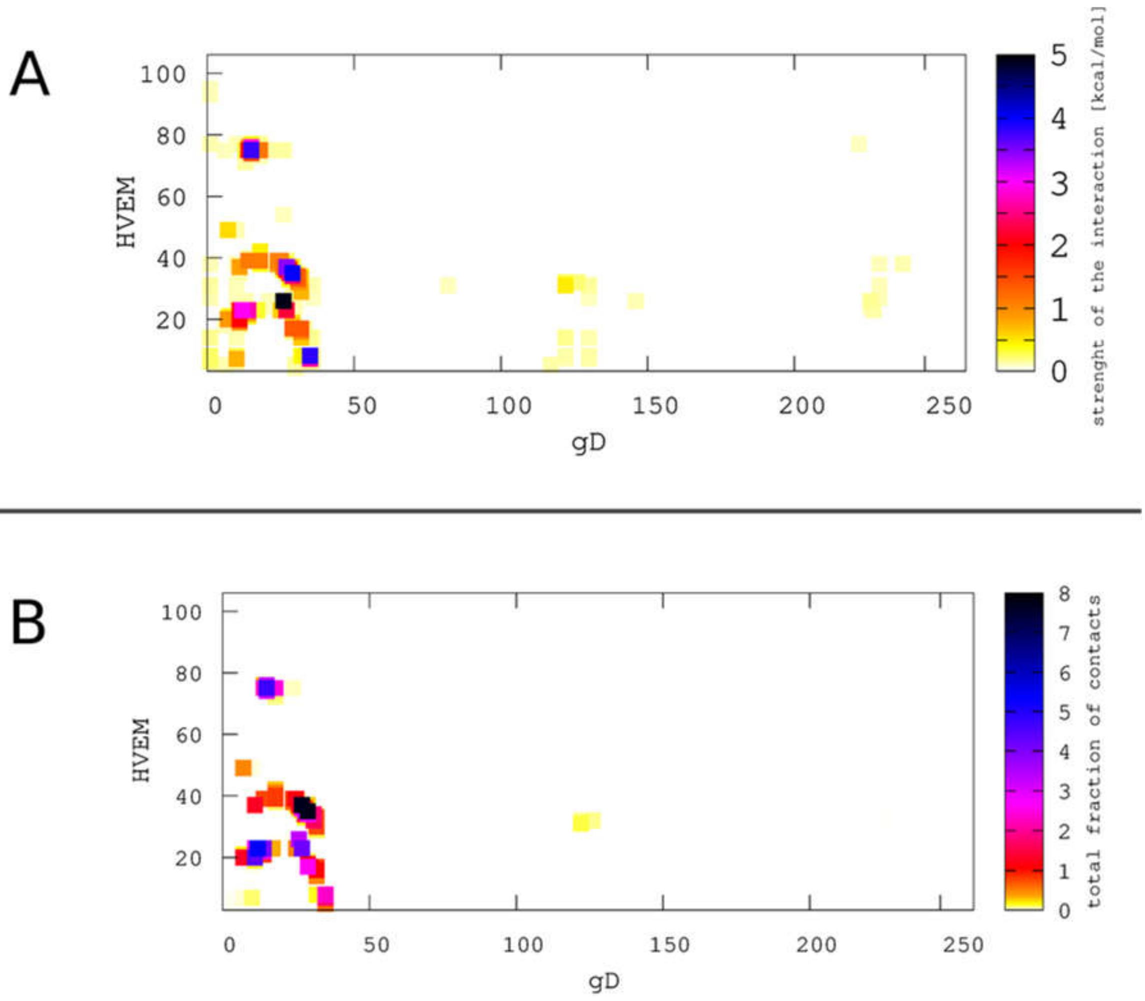

Figure S1. (A) interaction energies between gD and HVEM residues calculated by the energy decomposition on a pairwise per-residue level calculated using MM/GBSA analysis method. (B) the total fraction of contacts for the residue pair calculated using CPPTRAJ tool from AMBER package. The total fraction value is calculated for each pair as the sum of each contact involving that pair divided by the total number of frames, thus values can be greater than 1 if the residue pair includes more than 1 native contact.

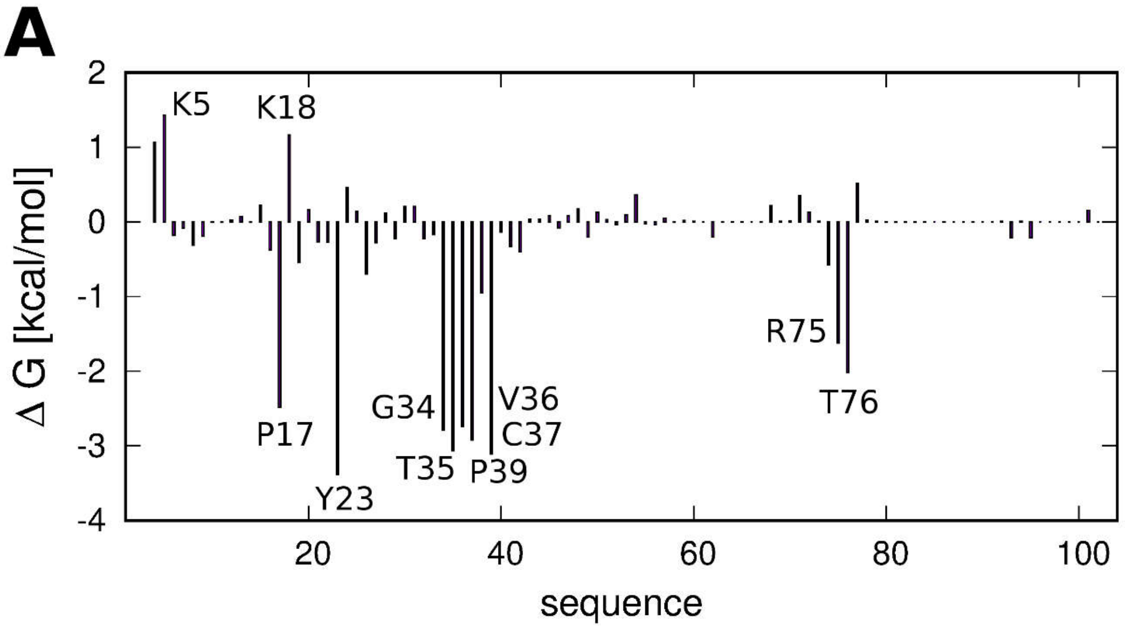

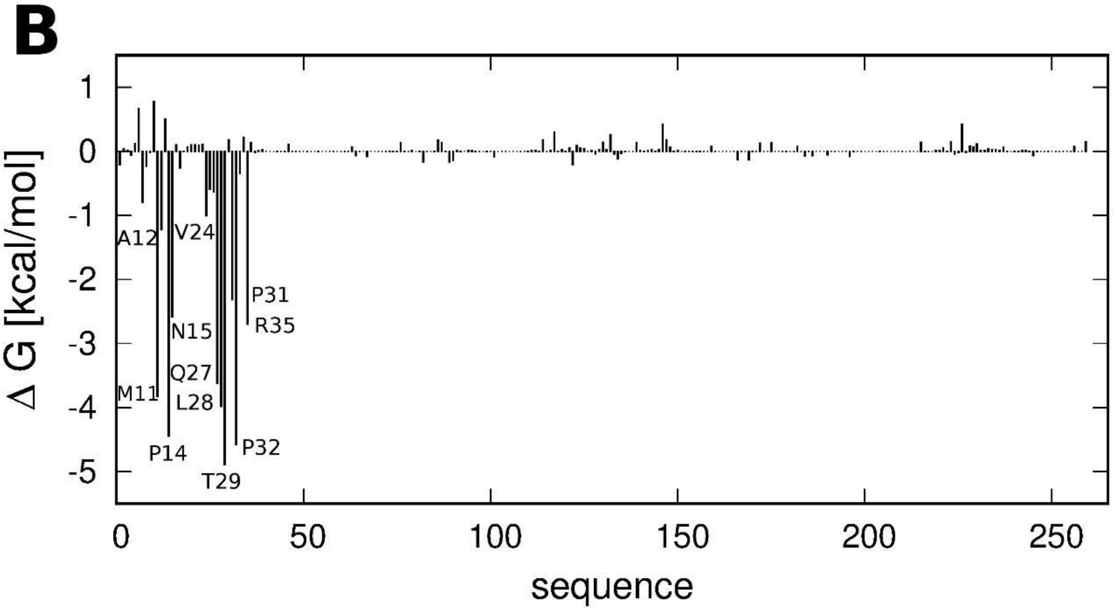

Figure S2. The energy decomposition on per-residue basis for HVEM (A) and gD (B) amino acid residues.

Table S1. Description of the strongest interactions between gD and HVEM residues assessed based on the energy decomposition on a pairwise per-residue level calculated using MM/GBSA analysis method.

Interaction energy	HVEM		gD	
[kcal/mol]	RName	RNum	RName	RNum
-4.913	LYS	26	ASP	26
-4.012	THR	35	THR	29
-3.816	GLU	8	ARG	35
-3.762	ARG	75	ASN	15
-3.432	CYS	37	GLN	27
-2.959	TYR	23	ALA	12
-2.818	THR	76	ASN	15
-2.685	TYR	23	PRO	14
-2.644	ASP	7	ARG	35
-2.454	GLY	34	THR	29
-2.373	THR	35	LEU	28
-2.241	TYR	23	GLN	27
-2.080	VAL	26	GLN	27
-2.043	ARG	75	PRO	14
-2.023	VAL	36	LEU	28
-2.015	SER	20	MET	11
-1.972	TYR	23	MET	11
-1.503	SER	74	ASN	15
-1.397	GLY	34	ASP	30
-1.352	PRO	17	PRO	32
-1.347	PRO	17	THR	29
-1.281	THR	33	PRO	31
-1.240	GLY	34	PRO	31
-1.213	CYS	37	ASP	26
-1.144	ARG	75	ARG	18
-1.113	PRO	39	PRO	14
-1.097	PRO	39	ARG	18
-1.060	CYS	19	MET	11
-1.056	PRO	39	VAL	24
-1.056	PRO	39	LEU	25
-1.023	CYS	16	PRO	32

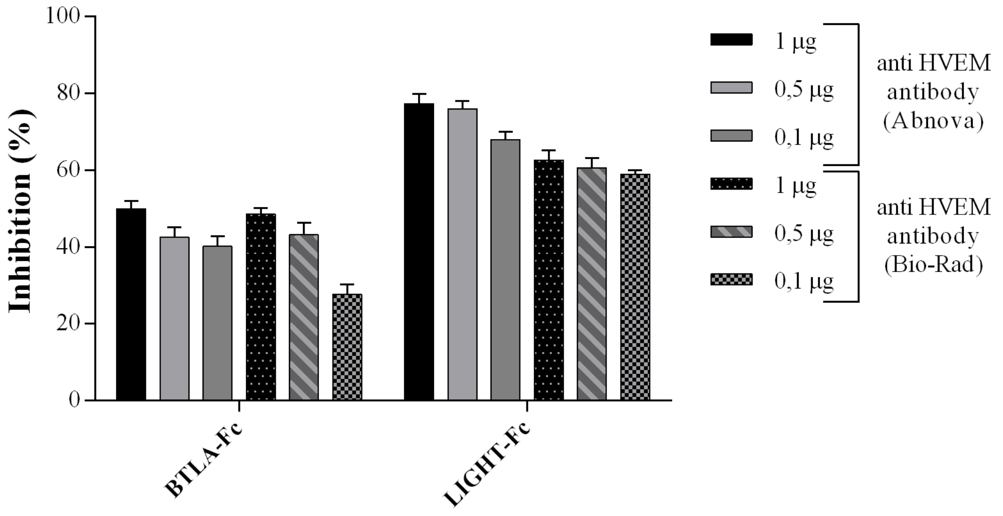

Figure S3. The inhibitory properties of the peptides were compared with two different commercially available anti-HVEM antibodies. Three different concentrations of HVEM antibodies were tested: 0.1, 0.5 and 1 µg/well. The obtained data show that the antibodies have similar inhibitory properties and inhibit the BTLA/HVEM complex formation in about 50% and HVEM/LIGHT ligation in about 75%.

Table S2. Amino acids sequences of gD(1-36)(L10C-T29C) and gD(1-36)(L10C-T29C)^{SCR} peptides.

Peptide name	Amino acid sequence
**gD(1-36)(K10C-T29C)**	Ac-KYALVDASLCMADPNRFRGKDLPVLDQLCDPPGVRR-NH_2
**gD(1-36)(K10C-T29C)^{scr}**	Ac-DRPLRASKGCDGVAVDLLDMPYPRQKNPCLVLDFAR-NH_2

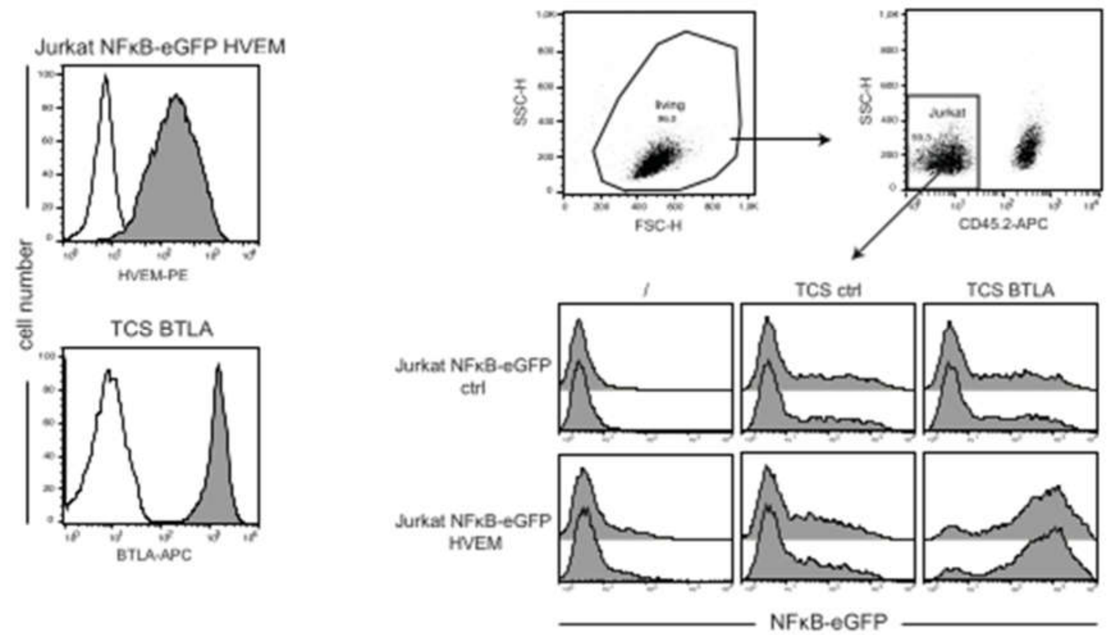

Figure S4. Evaluation of HVEM in a reporter based system. Left panel: Cell surface expression of the indicated molecules on Jurkat NFκB-eGFP reporter cells and T cell stimulator cells (TCS) analysed via flow cytometry. (Open histograms: control cells; grey histograms: expression level of the indicated molecules). Right panel: Gating strategy of one representative experiment. Control Jurkat NFκB-eGFP cells and Jurkat NFκB-eGFP expressing HVEM were left unstimulated or stimulated with control TCS or TCS expressing BTLA. TCS were excluded by using a mouse CD45.2 antibody and NFkB-eGFP expression was measured via flow cytometry. Histograms show NFκB-eGFP activation.

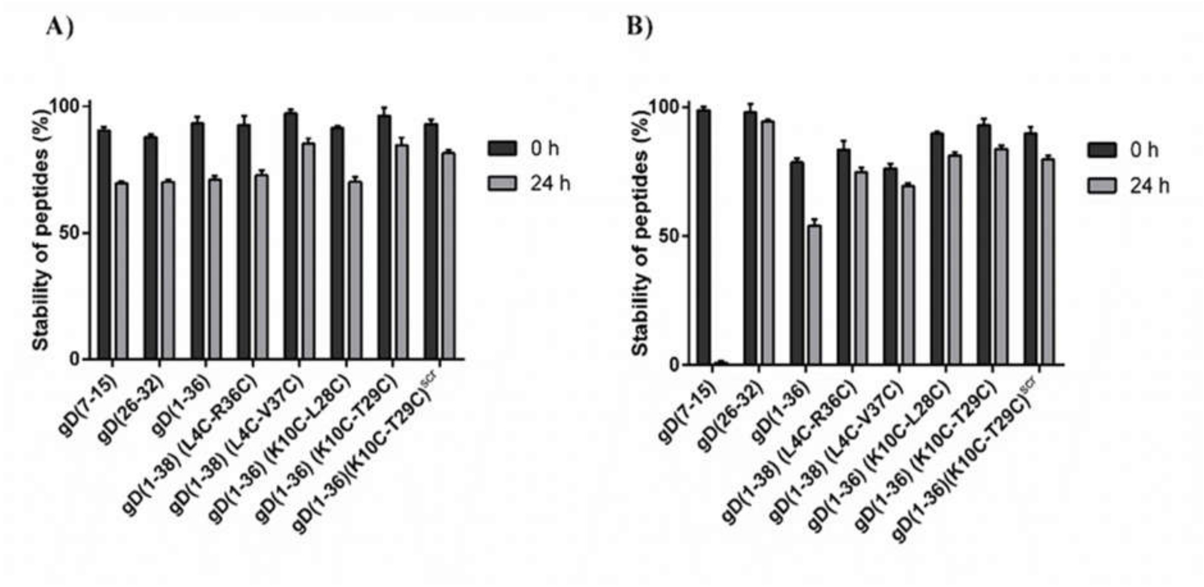

Figure SS5. The peptides stability (mean ± SEM) in A) PBS buffer, B) medium. The graphs show percentage of peptides remaining in the sample based on RP-HPLC data.

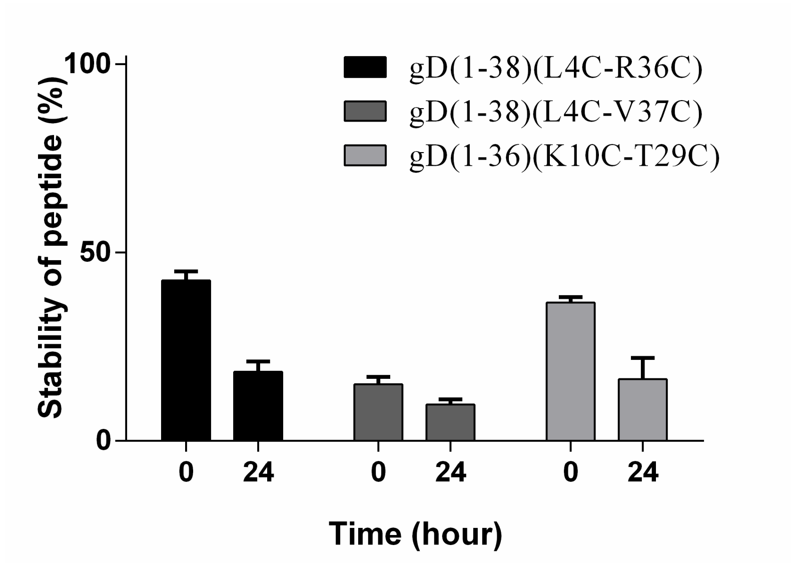

Figure SS6. The peptides stability (mean ± SEM) in plasma. The graph shows percentage of peptide remaining in the sample based on RP-HPLC data.

A)

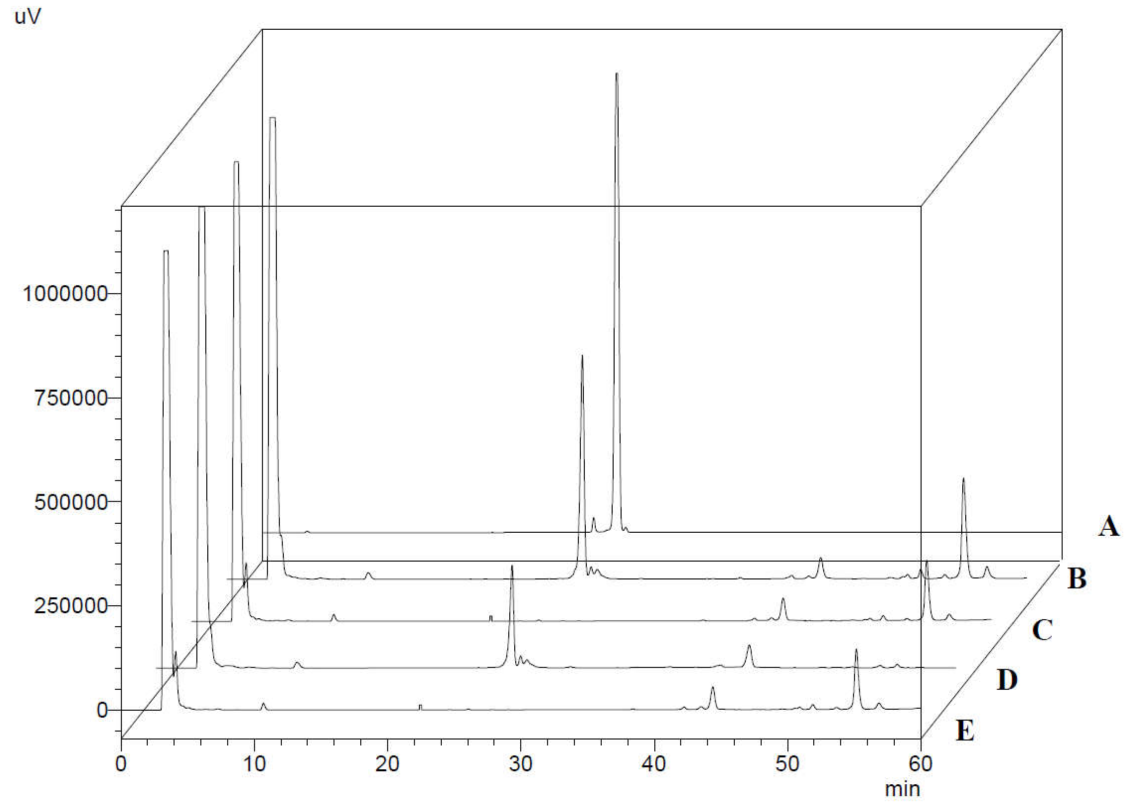
uV
1000000
750000
500000
250000
0
0
10
20
30
40
50
60
min
A
B
C
D
E

B)

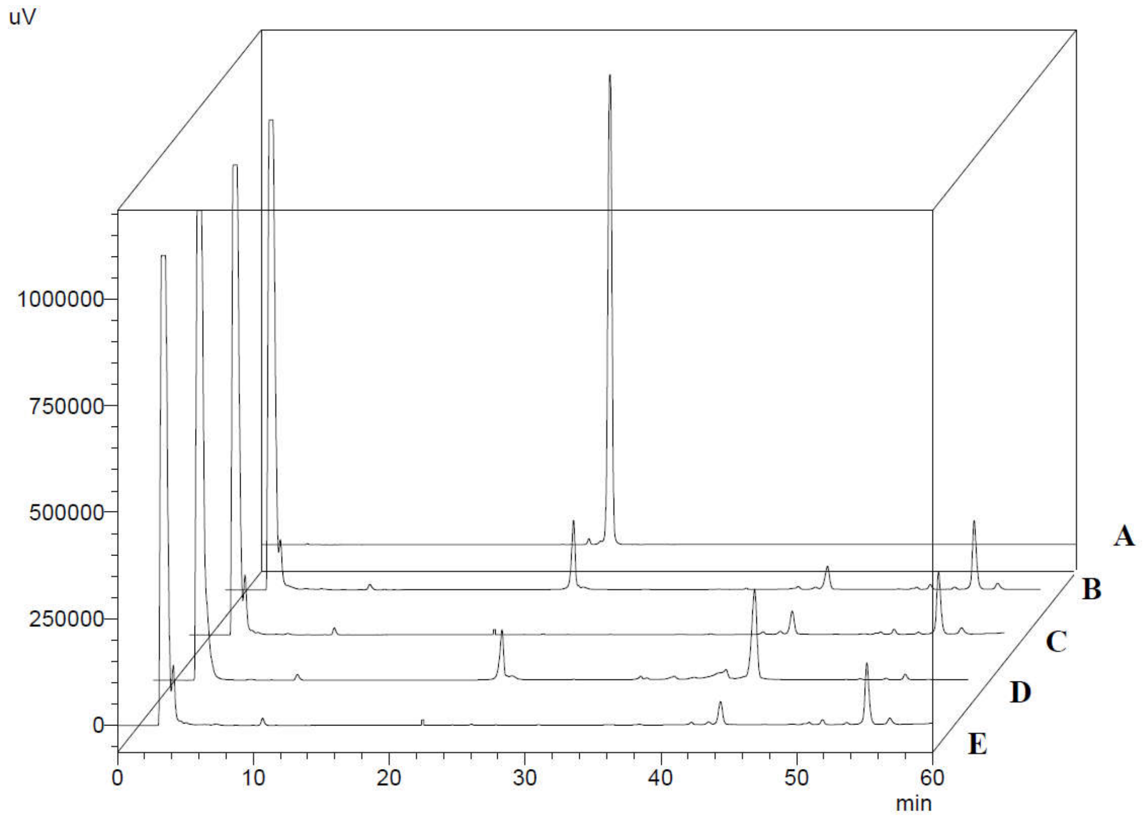
uV
1000000
750000
500000
250000
0
0
10
20
30
40
50
60
min
A
B
C
D
E

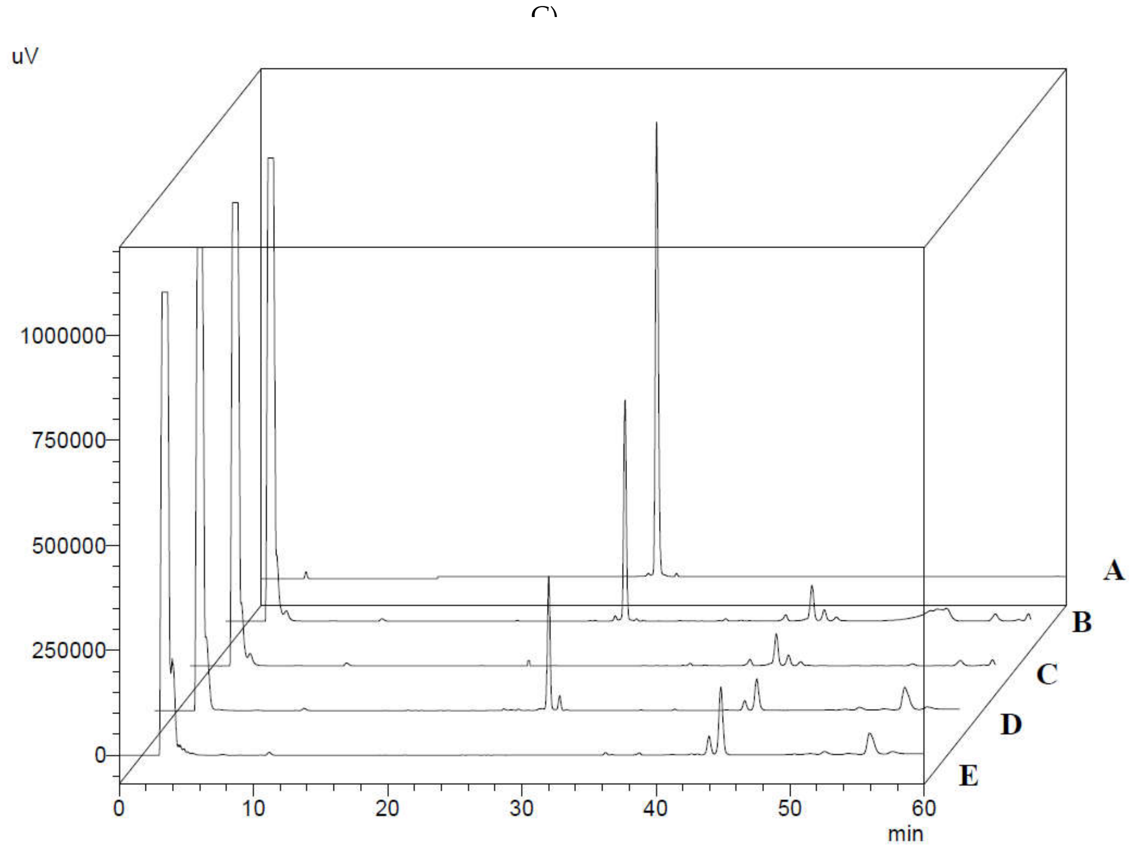

Figure S7. Chromatogram comparison of **A**) gD(1-38)(L4C-R36C), **B**) gD(1-38)(L4C-V37C) and **C**) gD(1-36)(K10C-T29C) peptides before incubation (**A**), peptide and plasma t=0 (**B**), plasma t=0 (**C**), peptide and plasma t=24 h (**D**), plasma t=24h (**E**).

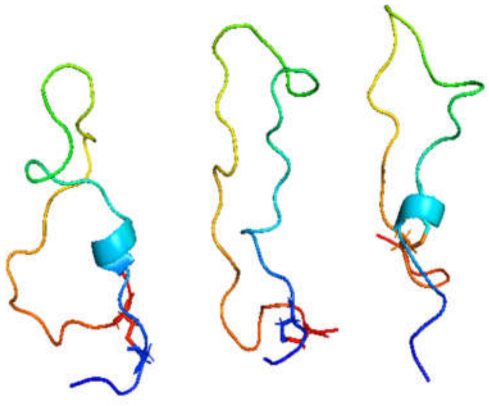

Figure S8. Structures of A) gD(1-38)(L4C-R36C), B) gD(1-38)(L4C-V37C) and C) gD(1-36)(K10C-T29C) obtained after all-atom simulations. The peptides are rainbow colored from N-terminal (blue) to C-terminal (red).
